# Robotic Tactile Sensing for Early Detection of Frost-Damaged Citrus Fruits with Pressure–Vibration Multimodal Fusion

**DOI:** 10.3390/foods15091597

**Published:** 2026-05-05

**Authors:** Yida Yu, Zihao Wu, Changqing An, Xiaopeng Lv, Yiran Zhao, Huirong Xu

**Affiliations:** 1 College of Biosystems Engineering and Food Science, Zhejiang University, Hangzhou 310058, China; ydyu2000@zju.edu.cn (Y.Y.);; 2 Zhejiang Key Laboratory of Intelligent Sensing and Robotics for Agriculture, Hangzhou 310058, China; 3 Key Laboratory of On-Site Processing Equipment for Agricultural Products, Ministry of Agriculture and Rural Affairs, Hangzhou 310058, China

**Keywords:** citrus frost damage, robotic sorting, multimodal tactile perception, pressure–vibration fusion, transformer, temporal attribution

## Abstract

Early-stage frost damage in citrus fruits is difficult to detect because external symptoms are often weak or absent, hindering intelligent robotic sorting in postharvest scenarios. To address this challenge, this study proposes a robotic multimodal tactile sensing approach inspired by human mechanoreception for frost-damage detection during grasping. A robotic gripper equipped with a 6×6 pressure matrix sensor and a piezoelectric vibration sensor was used to capture complementary tactile cues during standardized fruit handling, enabling the perception of subtle mechanical changes associated with early frost injury. Using 240 *Citrus reticulata* ‘Hong Mei Ren’ fruits under controlled experimental conditions, a Transformer-based multimodal fusion network was developed to jointly model pressure and vibration sequences for binary classification of normal and frost-damaged fruits. Across repeated stratified random-split experiments, the proposed method achieved a mean classification accuracy of 93.1%. Comparative experiments showed that the fusion model outperformed representative sequence-learning baselines, and ablation analysis confirmed that pressure–vibration fusion was more effective than either single modality alone. Attention-based temporal attribution further revealed that the most informative cues were concentrated in the initial contact and early loading stages, indicating the importance of early transient mechanical responses for frost-damage discrimination. Overall, the proposed approach demonstrates the feasibility of grasp-based robotic frost-damage detection under controlled experimental conditions.

## 1. Introduction

According to FAOSTAT, world citrus production reached 165.63 million tons in 2024 [1]. Among the quality defects affecting citrus fruits, frost damage is particularly problematic because it disrupts internal tissue structure, accelerates postharvest deterioration, and reduces commercial value [2,3,4,5]. Early frost damage often does not produce obvious external symptoms, making affected fruit difficult to identify reliably by appearance alone [6]. This mismatch between hidden internal damage and visually subtle symptoms poses a significant challenge for intelligent robotic sorting of citrus fruits [7,8,9].

As robots are increasingly deployed in postharvest handling, sorting, and packaging of fresh produce, they are expected not only to grasp and transfer fruit, but also to support quality-aware decision-making during manipulation [10,11]. For robotic fruit sorting, the key challenge is not only whether frost-damaged fruit can be identified, but whether quality information can be perceived directly during grasping. In practice, robotic sorting systems must localize, grasp, stabilize, and classify fruits in rapid succession under uncertainty in pose, size, curvature, and contact state. Existing robotic pipelines are still dominated by visual appearance and geometric cues, and therefore lack an effective in-gripper sensing mechanism for perceiving subtle internal quality defects during manipulation. This limitation is especially critical for early frost damage, whose visual manifestations are often weak or delayed.

Tactile sensing offers a natural route to address this gap because physical contact is already inherent to robotic grasping. Rather than treating grasping solely as an actuation step, tactile sensing enables the grasp itself to become a sensing event. Through contact-induced deformation, tactile signals can reveal mechanical responses associated with internal tissue condition, providing information that is difficult to obtain from vision alone. Recent studies have demonstrated the potential of robotic tactile sensing for fruit-related tasks such as firmness evaluation and non-destructive handling [12,13,14,15,16]. Nevertheless, most existing robotic fruit-sensing studies focus on relatively coarse attributes and often rely on a single sensing modality, which limits their ability to detect subtle early-stage frost damage. Moreover, relatively few studies have addressed the problem of in-gripper detection of subtle internal frost injury during robotic handling, where weak damage signatures must be inferred from short, contact-driven tactile sequences rather than from obvious external symptoms.

From a biological perspective, human mechanoreception integrates heterogeneous tactile cues across different temporal scales to perceive fine changes in object condition. Inspired by this principle, artificial tactile systems have begun to emulate mechanoreceptive sensing using hydrogel-based ionic transduction, biomimetic skins, and neural tactile architectures capable of capturing both sustained pressure and transient vibration information [17,18,19]. For frost-damaged citrus fruits, such a multimodal strategy is particularly relevant because freeze injury may alter not only the quasi-static load distribution during grasping, but also the dynamic mechanical response of the fruit. In this sense, pressure and vibration sensing can be viewed as complementary modalities: pressure sensing reflects distributed and relatively sustained contact responses, whereas vibration sensing captures transient dynamic interactions that may be sensitive to subtle structural changes caused by frost damage [20].

A key challenge, however, lies in how to effectively fuse these heterogeneous tactile signals. Pressure and vibration differ in physical meaning, temporal characteristics, and noise patterns, and simple feature concatenation may not be sufficient to capture their cross-modal dependencies [21,22,23]. Transformer-based models provide a promising solution because their attention mechanism can capture long-range temporal dependencies and flexible interactions across multiple data streams [23,24,25]. For robotic tactile perception, this capability is especially valuable because damage-related signatures may emerge only through coordinated interpretation of slowly varying pressure patterns and rapidly changing vibration responses over the course of a grasp. However, despite the rapid development of multimodal learning, the specific combination of gripper-integrated pressure–vibration tactile sensing and Transformer-based temporal fusion for early frost-damage detection in citrus fruits remains insufficiently explored.

To address these challenges, this study proposes a mechanoreception-inspired robotic multimodal tactile sensing approach for early detection of frost-damaged citrus fruits. Specifically, a gripper-integrated sensing system combining a pressure matrix sensor and a vibration sensor is developed to capture complementary tactile responses during fruit grasping. On this basis, a Transformer-based multimodal fusion network is constructed for frost-damage classification. The proposed approach is systematically evaluated through comparative experiments on normal and frost-damaged citrus fruits, with particular emphasis on the discrimination of early-stage damage. In addition, attention-based temporal attribution analysis is introduced to provide a time-resolved view of the model’s emphasis during grasping. Compared with existing studies that often rely on single-modality sensing or non-contact inspection, the proposed framework emphasizes in-gripper perception of subtle internal frost injury through the joint use of quasi-static pressure cues and transient vibration cues. The main contributions of this study are as follows: (1) a robotic multimodal tactile sensing framework integrating a 6×6 pressure matrix sensor and a piezoelectric vibration sensor is established for grasp-based frost-damage detection in citrus fruits; (2) a Transformer-based multimodal fusion network is developed to jointly model complementary quasi-static and dynamic tactile sequences; and (3) the proposed method is validated under a standardized robotic grasping protocol and further examined through temporal attribution analysis.

## 2. Materials and Methods

### 2.1. Overall Experimental Workflow

**Figure 1:**
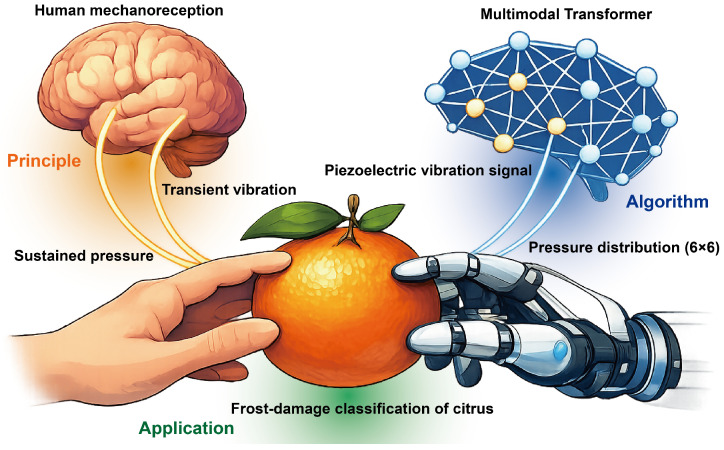
Overview of the proposed mechanoreception-inspired multimodal tactile workflow for citrus frost-damage detection.

Figure 1 presents the overall analytical framework of the proposed mechanoreception-inspired multimodal tactile method for citrus frost-damage detection. The workflow comprises four stages: synchronized multimodal tactile acquisition during robotic grasping, modality-specific preprocessing and fixed-length sequence construction, Transformer-based multimodal classification, and post hoc temporal attribution analysis. Inspired by the complementary encoding of sustained and transient tactile cues in human cutaneous mechanoreception, the robotic gripper integrates two synchronous sensing modalities: a 6×6 pressure matrix sensor that captures spatial pressure distributions during grasping, and a piezoelectric vibration sensor that records transient vibration signals induced by loading. Within this framework, the pressure modality provides quasi-static spatial contact information, whereas the vibration modality captures dynamic responses associated with contact onset and loading. This modality pairing is designed as a functional analogy to mechanoreceptive channels associated with sustained pressure and transient vibration.

During robotic grasping, pressure distributions and vibration signals are synchronously acquired and subsequently preprocessed to form multimodal time-series inputs. After preprocessing, the pressure and vibration sequences are standardized into fixed-length representations and used as the inputs to two dedicated Transformer branches, whose outputs are fused for frost-damage classification. These sequences are then used to train a Transformer-based fusion network in a supervised manner for frost-damage classification. In addition to quantitative evaluation, residual-aware self-attention analysis is performed to obtain attention-based temporal attribution profiles, which are visualized as heatmaps to indicate the time steps that contribute most to the model decision. This analytical framework links tactile data acquisition, sequence representation, multimodal learning, and temporal attribution analysis within a unified pipeline for grasp-based frost-damage detection.

### 2.2. Citrus Samples

A total of 240 *Citrus reticulata* ‘Hong Mei Ren’ fruits were hand-picked from Xiangshan County, Ningbo, Zhejiang Province, China. To reduce inter-sample variability, fruits were preselected for comparable maturity and external appearance. Fruit diameter ranged from 66.31 to 81.34 mm (73.54 ± 3.41 mm, mean ± SD), and mass ranged from 136.7 to 227.9 g (185.01 ± 23.56 g). After harvest, all samples were stored in a temperature- and humidity-controlled storage facility at the School of Biosystems Engineering and Food Science, Zhejiang University (15 °C, 75–85% relative humidity) until testing. Each fruit was individually numbered for tracking throughout the experiment.

A single-cultivar setting was adopted to enable controlled evaluation of the proposed sensing-and-learning pipeline under a practically relevant postharvest scenario. *Citrus reticulata* ‘Hong Mei Ren’ was selected because it is a commercially important cultivar in Zhejiang Province and is susceptible to frost-related quality defects during the harvest season.

To prepare samples with controlled frost injury, half of the fruits (*n* = 120) were exposed to −5 °C for 12 h in a programmable low-temperature chamber, whereas the other half (*n* = 120) were kept as untreated controls [3,26]. After treatment, all fruits were equilibrated for 24 h at 20–25 °C before robotic grasping. All tactile measurements were collected after equilibration to minimize confounding effects caused by transient temperature differences. The experimental campaign was conducted from 6 January to 20 January 2025.

Ground-truth labels were obtained by destructive post-measurement inspection. After robotic tactile data acquisition, each fruit was cut open and its internal tissue condition was visually examined. Fruits exhibiting internal freeze-injury symptoms, such as water-soaked, translucent, or structurally degraded pulp tissue, were labeled as frost-damaged, whereas fruits without such symptoms were labeled as normal.

### 2.3. Robotic Platform and Mechanoreception-Inspired Multimodal Tactile Sensing

**Figure 2:**
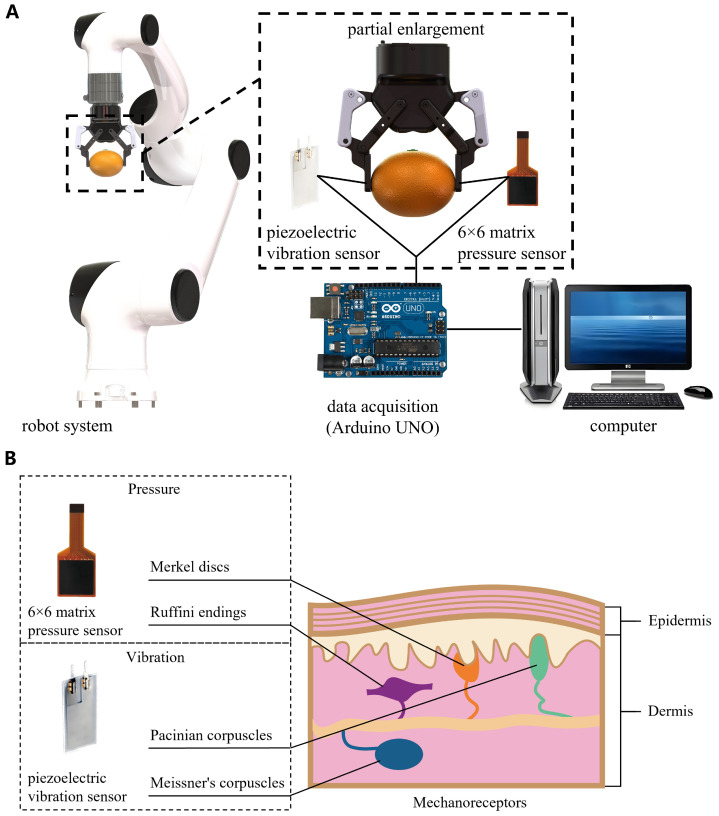
(**A**) Robotic platform for frost-damage detection in citrus fruits. (**B**) Conceptual mapping between representative cutaneous mechanoreceptors, their stimulus sensitivities, and the sensing modalities.

The robotic sorting platform was developed for postharvest indoor quality inspection and sorting under controlled facility conditions [11]. In this setting, fruits are typically processed on grading lines under relatively stable environmental and operating conditions, making it suitable for evaluating the proposed sensing system [27,28]. As shown in Figure 2A, the platform integrates a two-finger gripper (Robotiq 2F-85 Gripper, Robotiq Inc., Lévis, QC, Canada) mounted on a robotic arm (Elfin E10-Pro, Shenzhen Han’s Robot Co., Ltd., Shenzhen, China). The gripper provides programmable control of grasping force, closing speed, and finger position, with a force range of 20–235 N, a closing speed range of 20–150 mm/s, and a position repeatability of 0.05 mm. By regulating these grasping parameters, the system enables stable and gentle handling of citrus fruits during robotic sorting.

To enable multimodal tactile sensing, a 6×6 pressure matrix sensor (RPPS-36, LEGACT, Hong Kong, China) was mounted on one gripper finger to capture spatially resolved contact pressure distributions, while a polyvinylidene fluoride (PVDF) piezoelectric film sensor (LDT0-028K, TE Connectivity Measurement Specialties, Plymouth, MN, USA) was attached to the opposing finger to record dynamic vibration responses during contact loading. The robotic arm and gripper were controlled by a host computer to execute grasping motions while synchronously recording pressure and vibration signals during contact. The pressure matrix sensor and vibration sensor were connected to their respective signal-conditioning circuits and interfaced with an Arduino Uno R3 (Arduino, Monza, Italy) for data acquisition. The acquired signals were then transmitted to the host computer for subsequent processing and analysis.

This study adopts a mechanoreception-inspired multimodal tactile sensing scheme that combines the pressure matrix sensor and the piezoelectric vibration sensor mounted on the robotic gripper. The design is motivated by the complementary encoding of quasistatic and dynamic tactile cues in human cutaneous mechanoreception [29], and aims to capture subtle mechanical changes associated with frost-induced tissue degradation during grasping [30]. In the human somatosensory system, Merkel discs and Ruffini endings are commonly associated with sustained pressure and skin deformation, whereas Meissner’s corpuscles and Pacinian corpuscles are more sensitive to transient vibration and dynamic touch stimuli [18].

As illustrated in Figure 2B, this biological analogy motivates the pairing of two complementary sensing modalities. Specifically, the 6×6 matrix pressure sensor provides a spatial map of contact pressure during grasping, reflecting changes in contact compliance and load distribution associated with frost-induced tissue alteration [31]. The piezoelectric vibration sensor records vibration signatures generated during contact loading, which may vary with the internal texture and mechanical integrity of the fruit tissue [19]. Together, these modalities provide complementary information: pressure distribution as a quasi-static cue and vibration as a dynamic cue, thereby supporting the detection of early-stage frost damage even when external visual symptoms are absent.

### 2.4. Sensor Data Acquisition and Preprocessing

The control logic of the robotic platform positions each citrus fruit centrally between the two parallel fingers at each grasp, enabling stable and repeatable manipulation. The gripper operates under closed-loop control of finger position, speed, and gripping force. During each trial, the fingers close at a preset speed of 50 mm/s until the measured gripping force reaches a predefined maximum of 20 N, after which finger motion is halted and the grasp is maintained for 5 s. The gripper then opens, and data acquisition is terminated.

The pressure and vibration modalities were synchronously acquired through the Arduino Uno R3-based data acquisition system during the same grasping event. Based on the recorded raw timestamps, the effective sampling rates were approximately 20 Hz for the pressure modality and approximately 200 Hz for the vibration modality. For multimodal sequence preparation, both signals were segmented according to the same grasping trial and subsequently standardized into fixed-length model inputs before being fed into the network.

**Figure 3:**
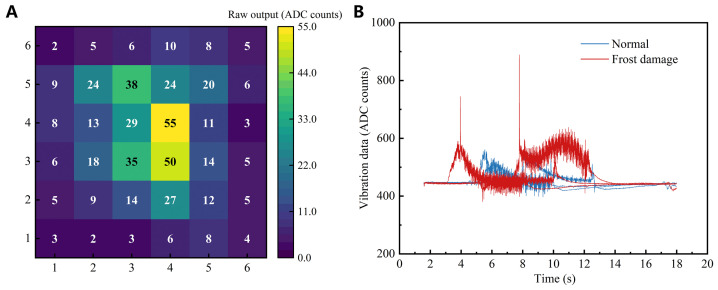
Representative raw tactile signals acquired during robotic grasping. (**A**) Pressure matrix signal. (**B**) Vibration sensor signal.

The pressure modality provides a time sequence of 6×6 readings that reflects the spatial distribution and temporal evolution of contact pressure during grasping [32]. The raw pressure signals (Figure 3A) were recorded as 10-bit ADC counts (0–1023). Because the raw pressure and vibration streams had different effective sampling rates, temporal correspondence was established at the grasp-event level rather than by direct one-to-one matching of raw samples. Preprocessing included temporal denoising, sequence-length standardization of each grasping trial to a fixed model-input length of 50 time steps, and sample-wise normalization of the matrix sequence to reduce inter-trial scale variability [33,34]. Thus, the 50 time steps used in the model refer to the preprocessed temporal representation after standardization, rather than to the raw sequence length acquired during sensing.

The vibration modality records time-domain responses during loading (Figure 3B), which were also digitized as 10-bit ADC counts (0–1023). To emphasize dynamic components associated with transient events and suppress quasi-static drift, the vibration signal was first filtered using a second-order Butterworth high-pass filter with a cutoff frequency of 30 Hz to attenuate low-frequency components introduced by the grasping process [35]. The filtered vibration signal was then standardized to the same fixed model-input length of 50 time steps and used directly as the temporal input to the vibration branch of the model.

### 2.5. Transformer-Based Multimodal Fusion Network

**Figure 4:**
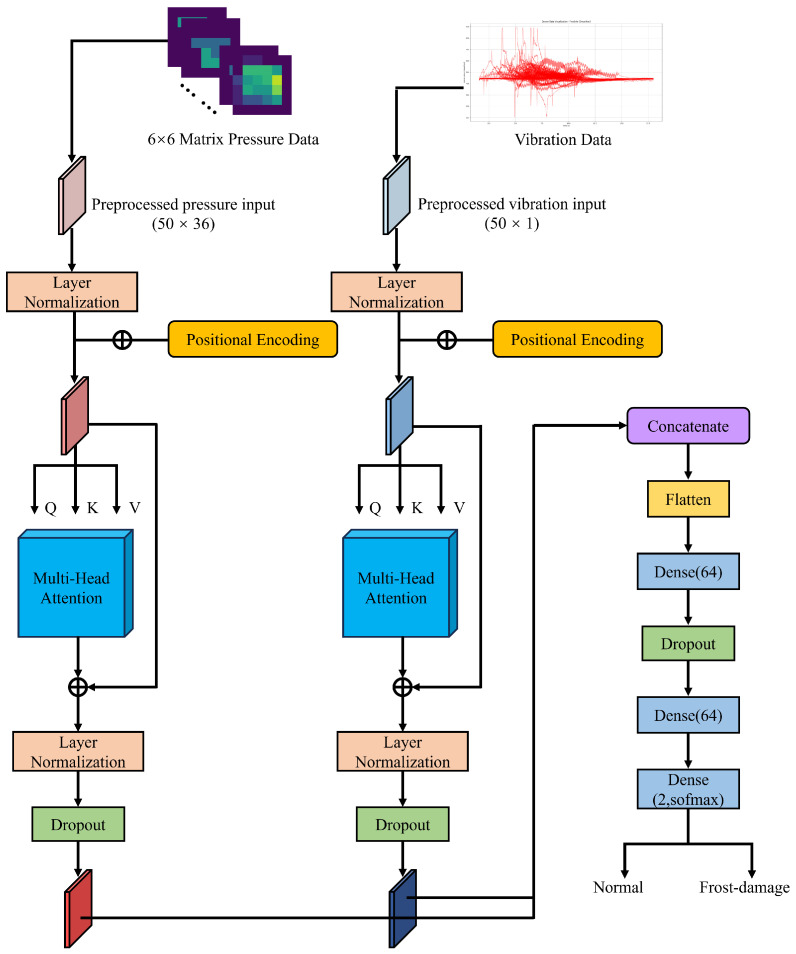
Architecture of the proposed Transformer-based multimodal fusion network.

As shown in Figure 4, the proposed architecture comprises four stages: modality-specific input formation, branch-wise Transformer encoding, multimodal feature fusion, and final classification. A Transformer-based multimodal fusion network was developed to integrate pressure-matrix and vibration signals for frost-damage classification [23]. The pressure sequence and vibration sequence were treated as two temporally aligned input modalities with shapes of 50×36 and 50×1, respectively, and were processed by dedicated Transformer branches to learn modality-specific representations. The pressure input was represented as a sequence of 50 time steps, each associated with a 36-dimensional feature vector obtained by flattening the 6×6 pressure matrix, whereas the vibration input was represented as a sequence of 50 scalar values. No separate learnable embedding layer was introduced before the Transformer branches. Instead, the modality-specific inputs were first normalized and then combined with fixed positional encoding to preserve temporal-order information before self-attention. The subsequent linear projections used to form the query (*Q*), key (*K*), and value (*V*) representations were learned internally within each multi-head self-attention module. Each branch contained one self-attention block consisting of layer normalization, multi-head self-attention, a second layer normalization, and dropout. Each modality branch therefore functioned as a single-block Transformer encoder. The multi-head attention module used two attention heads with a key dimension of 32, and the dropout rate was set to 0.3. The outputs of the two modality-specific branches were then concatenated and flattened, and the fused representation was passed through a classification head composed of a fully connected layer with 64 units, a dropout layer, a second fully connected layer with 64 units, and a final softmax output layer with two units to predict whether a fruit was normal or frost-damaged.

As shown in Figure 4, the parallel Transformer branches are used to model dependencies within each modality, while the fusion module combines complementary information from pressure and vibration signals. The pressure branch was designed to capture the temporal evolution of distributed quasi-static contact patterns, whereas the vibration branch was used to encode transient dynamic tactile responses during grasping. For the pressure modality, each time step therefore entered the branch as a 36-dimensional vector corresponding to the flattened 6×6 pressure matrix, whereas for the vibration modality, each time step entered as a scalar value. In both branches, temporal interactions were modeled after positional encoding through the internally learned *Q*/*K*/*V* projections and self-attention operation. Through the self-attention mechanism, the network can assign different weights to informative patterns in the input sequences, thereby supporting multimodal feature extraction for classification. After branch-wise encoding, the modality-specific outputs were concatenated at the representation level and then passed to the classification head for multimodal integration and prediction. Compared with direct feature concatenation at the raw-input level, this branch-wise design allows each modality to first form its own temporal representation before multimodal fusion, thereby improving the modeling of cross-modal complementarity. Each branch employed two attention heads, from which head-specific attention weights could be extracted separately; the corresponding head-level attention analysis and aggregation procedure are described in Section 2.7.

### 2.6. Model Training Settings

Unless otherwise stated, the baseline and ablation models were trained using the same optimization settings for fair comparison. The preprocessed sequences described in Section 2.4 were divided into training, validation, and test sets at a ratio of 7:2:1 using stratified sampling with different random seeds for repeated evaluation rather than relying on a single fixed split. Specifically, the random partitioning procedure was repeated 10 times, and the corresponding evaluation results were summarized as mean ± standard deviation. Ground-truth labels were converted to one-hot vectors for two-class classification. No data augmentation or class reweighting was applied. To ensure a fair comparison, all competing models were trained under the same preprocessing pipeline, split ratio, optimization settings, and evaluation protocol. The main hyperparameters were selected empirically based on validation-set performance.

Model optimization was performed using the Adam optimizer with an initial learning rate of 0.001 and a batch size of 16. The maximum number of training epochs was set to 300. To reduce overfitting, early stopping was applied by monitoring the validation loss with a patience of 20 epochs, and the model weights corresponding to the best validation performance were restored. Validation loss was evaluated after each epoch, and training was terminated when no improvement was observed for 20 consecutive epochs. For each repeated run, the checkpoint corresponding to the minimum validation loss was retained and restored for the subsequent evaluation. The proposed model was implemented in a Conda-managed Python environment using TensorFlow v2.16.1 with CUDA acceleration and trained on a Linux server equipped with an Intel(R) Xeon(R) Silver 4210 CPU @ 2.20 GHz, 62 GB RAM, and a single NVIDIA A10 GPU (23 GB memory) for training and inference. The proposed Transformer-based fusion model contained 35,931 trainable parameters. Across repeated runs, the model typically converged under the early-stopping criterion well before the maximum epoch limit. During prediction, inference on the held-out test set was completed in two steps with an average runtime of approximately 109 ms per step, corresponding to a total prediction time of about 0.22 s in a representative run. These results indicate that the network itself was computationally lightweight relative to the grasp duration used in this study. Nevertheless, full real-time deployment in industrial sorting lines would still require further system-level validation including sensing, communication, and robotic actuation delays.

### 2.7. Attention-Based Temporal Attribution and Heatmap Visualization

#### 2.7.1. Self-Attention Maps

To improve the interpretability of the multimodal Transformer classifier, attention maps were analyzed to summarize the temporal emphasis of the model during sequential tactile processing [25,36]. The resulting attribution patterns provide a post hoc view of model emphasis and should be interpreted as correlational rather than causal. In the present network, each modality branch contains one multi-head self-attention module with two attention heads. Therefore, for each input sample and for each modality branch, two head-specific attention matrices are first obtained before branch-level aggregation.

For each modality branch, the multi-head self-attention module computes attention weights from query–key similarity according to [24]

(1) $$A^{\left(h\right)}=\mathrm{softmax}\;\left(\frac{Q^{\left(h\right)}{K^{\left(h\right)}}^{⊤}}{\sqrt{d_{k}}}\right),$$

where h∈{1,…,H} denotes the attention-head index, *H* is the total number of attention heads, and $d_{k}$ is the key dimension. For a sequence of length *T*, the resulting attention matrix $A^{\left(h\right)}\in R^{T\times T}$ contains entries $A_{t,t^{\prime }}^{\left(h\right)}$, where *t* denotes the query time step and *t*’ denotes the key/value time step. Thus, $A_{t,t^{\prime }}^{\left(h\right)}$ represents the attention weight assigned by query position *t* to position empht’. Here, each matrix $A^{\left(h\right)}$ serves as the head-level attention map for head emphh within the corresponding modality branch. In this study, head-level attention analysis refers to first computing these per-head temporal attention maps and then aggregating them within each branch to obtain a branch-wise summary of temporal emphasis. Because the present analysis focuses on the overall temporal emphasis of each modality branch rather than on functional specialization of individual heads, the head-specific maps were averaged before subsequent rollout analysis. Multi-head attention maps are aggregated by averaging across heads:

(2) $$\bar{A}=\frac{1}{H}\sum_{h=1}^{H}A^{\left(h\right)},$$

where the averaging operation removes the explicit head dimension. The resulting matrix $\bar{A}$ therefore represents the branch-level temporal interaction pattern jointly summarized from the two attention heads. Accordingly, the subsequent matrices $\bar{A}$, $\tilde{A}$, and *R* are defined over temporal positions only and no longer carry a head index.

#### 2.7.2. Residual-Aware Attention Rollout and Temporal Attribution Score

To account for residual connections around the attention sub-layer, a residual-adjusted attention map is defined as [37,38]

(3) $$\tilde{A}=\mathrm{RowNorm}\left(\bar{A}+I\right),$$

where *I* is the identity matrix and RowNorm(·) denotes row-wise normalization such that each row sums to one.

When multiple self-attention layers are stacked, attention rollout propagates attention through layers by matrix multiplication:

(4) $$R={\tilde{A}}^{\left(1\right)}{\tilde{A}}^{\left(2\right)}\cdots {\tilde{A}}^{\left(L\right)}.$$

Here, *L* denotes the number of stacked self-attention layers in one modality branch. In the present architecture, each modality branch employs a single self-attention block; therefore, *L* = 1 and the rollout reduces to the residual-adjusted attention map, i.e., $R=\tilde{A}$. This formulation is retained for consistency with the general rollout definition. Accordingly, the subsequent temporal attribution analysis was performed on the head-averaged branch-level attention map after residual adjustment, rather than on separate head-wise visualizations.

Because no dedicated [CLS] token is used, a scalar temporal attribution score for each time step is defined as the average incoming attention over all query positions:

(5) $$s\left(t\right)=\frac{1}{T}\sum_{q=1}^{T}R_{q,t},$$

where *q* indexes the query position and $R_{q,t}$ denotes the element in row *q* and column *t* of the rollout matrix *R*, i.e., the aggregated attention from query position *q* to time step *t*. Accordingly, s(t) measures how strongly time step *t* is attended to, on average, by all query positions in the sequence. The score s(t) is computed independently for the pressure branch and the vibration branch, yielding modality-specific temporal attribution profiles.

#### 2.7.3. Heatmap Normalization and Peak-Time Statistics

For visualization, the temporal attribution score s(t) of each sample was normalized to the range [0,1] using min–max normalization:

(6) $$\hat{s}\left(t\right)=\frac{s\left(t\right)-\min_{t}s\left(t\right)}{\max_{t}s\left(t\right)-\min_{t}s\left(t\right)+\epsilon },$$

where ϵ is a small constant added to avoid division by zero. The normalized scores ${\hat{s}}_{p}\left(t\right)$ and ${\hat{s}}_{v}\left(t\right)$ were first obtained for the pressure and vibration branches, respectively.

For sample-level visualization, the two modality-specific attribution profiles were combined by equal-weight averaging:

(7) $${\hat{s}}_{\mathrm{fused}}\left(t\right)=\frac{1}{2}\left({\hat{s}}_{p}\left(t\right)+{\hat{s}}_{v}\left(t\right)\right),$$

where ${\hat{s}}_{p}\left(t\right)$ and ${\hat{s}}_{v}\left(t\right)$ denote the normalized attribution scores of the pressure and vibration branches, respectively. The fused score ${\hat{s}}_{\mathrm{fused}}\left(t\right)$ was used to generate a single temporal attribution heatmap for each sample. At the sample level, the fused visualization summarizes the overall temporal concentration of multimodal model emphasis, while the pressure and vibration branches retain their own modality-specific attribution profiles.

To summarize the dominant attention timing, the peak time step was defined as

(8) $$t^{*}=\arg\max_{t}{\hat{s}}_{\mathrm{fused}}\left(t\right),$$

where $t^{*}$ denotes the time step with the highest fused normalized attribution score. For statistical analysis, the peak time steps were grouped into time intervals, and the frequency distribution of these peak intervals was summarized as a histogram. Peak-time statistics were computed using all samples and were used to compare the temporal concentration of attention across classes.

## 3. Results and Discussion

### 3.1. Comparison of Network Architectures

To evaluate the effectiveness of the proposed multimodal Transformer architecture, its classification performance was compared with three commonly used sequence-learning baselines: recurrent neural network (RNN), convolutional neural network (CNN), and long short-term memory (LSTM). To reduce the influence of a potentially favorable or unfavorable single data split, the comparison was further extended to repeated random-split experiments. Specifically, the dataset was divided into training, validation, and test sets at a ratio of 7:2:1 using stratified sampling, and this procedure was repeated 10 times with different random seeds. All models were trained under the same preprocessing pipeline, optimization settings, and evaluation protocol, and the final results are reported as mean ± standard deviation on the test sets. As shown in Table 1, the Transformer consistently achieved the best overall performance among the compared architectures.

In addition to higher overall accuracy, the Transformer exhibited a more balanced classification performance across both classes. This result suggests that the proposed task benefits from a modeling framework that can simultaneously preserve temporal dependencies and flexibly emphasize informative stages within the grasping sequence. Compared with the recurrent and convolution-based baselines, the Transformer is better suited to handling multimodal tactile data in which useful discriminative cues may appear at different time steps and may not follow a strictly local or sequential pattern.

From a sensing perspective, frost-damage-related differences are likely reflected not only in the final contact state, but also in the temporal evolution of pressure redistribution and vibration response during grasping. Such cues are distributed across the sequence and may depend on interactions between relatively sustained and transient tactile signals. The self-attention mechanism provides a natural advantage in this setting because it allows the model to adaptively focus on informative temporal segments and to better capture long-range dependencies within each modality.

These results indicate that the Transformer is more effective at capturing discriminative temporal patterns in the multimodal tactile signals. Moreover, the repeated-run comparison indicates that the observed performance advantage was not merely caused by a particular random partition of the dataset, but reflects a relatively robust modeling benefit under the present experimental setting. Overall, the comparison supports the use of attention-based temporal modeling for early frost-damage detection.

### 3.2. Influence of Sensor Modalities on Model Performance in the Ablation Study

To assess the contribution of each sensing modality and the benefit of multimodal fusion, an ablation study was conducted on the proposed Transformer architecture [39]. The model integrates two complementary tactile modalities, namely piezoelectric vibration signals and 6×6 pressure matrix sequences. For fair comparison, the single-modality variants used the same backbone and training protocol as the multimodal model, with the other modality removed. In the single-modality settings, only the corresponding branch was retained and the fusion module was omitted.

Three input configurations were evaluated: vibration only, pressure only, and vibration + pressure fusion. To avoid bias caused by a single favorable training/validation/test split, the ablation results were also evaluated over 10 repeated stratified random splits with different random seeds, and the final metrics are reported as mean ± standard deviation on the test sets. As shown in Table 2, the fusion model achieved the best overall performance, followed by the pressure-only model, whereas the vibration-only model showed the lowest performance among the three configurations. These results indicate that both modalities contribute useful information, while pressure provides stronger discriminative capability than vibration when used alone.

Compared with the single-modality settings, the multimodal fusion model showed the most balanced class-wise performance across both normal and frost-damaged fruits over repeated runs. The stronger performance of the pressure-only model relative to the vibration-only model suggests that distributed contact-pressure patterns carried more discriminative information under the present experimental setting. Meanwhile, the further improvement achieved by multimodal fusion indicates that transient dynamic tactile responses still contributed useful supplementary cues beyond quasi-static pressure patterns. Overall, the ablation results further support the use of multimodal pressure–vibration fusion for early frost-damage detection.

### 3.3. Training Behavior and Generalization Assessment

**Figure 5:**
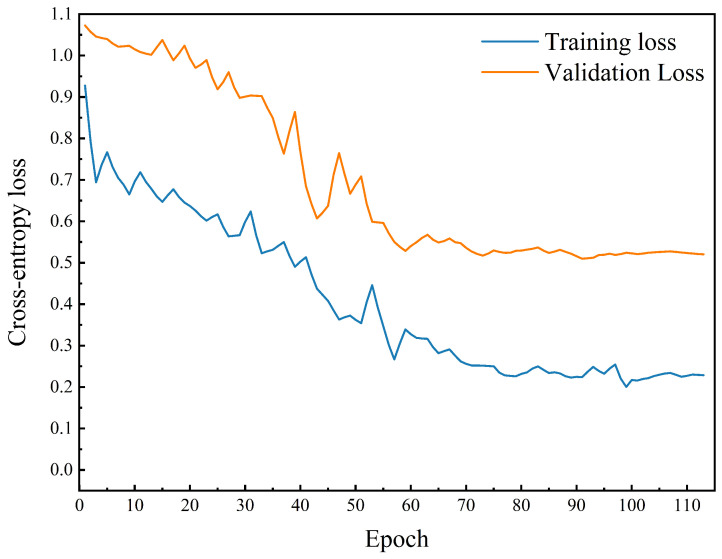
Training and validation cross-entropy loss curves of the proposed Transformer-based multimodal fusion model from a representative repeated-split run.

To further assess the training behavior and generalization characteristics of the proposed Transformer-based multimodal fusion model, the training and validation loss curves from a representative repeated-split run are shown in Figure 5. Overall, the training loss decreased progressively with increasing epochs, while the validation loss declined during the main training stage and then remained within a relatively limited fluctuation range near convergence. This behavior indicates that the adopted optimization strategy enabled effective convergence under the present experimental setting. Early stopping based on validation loss further helped prevent unnecessary continued training after performance ceased to improve, thereby supporting stable generalization performance.

Although some fluctuation in the validation curve is expected given the limited dataset size, the overall relationship between the training and validation losses suggests that the model maintained reasonably stable generalization during training, without severe divergence between training and validation losses. This observation is consistent with the use of regularized optimization and early stopping, which together helped the model achieve a favorable balance between fitting ability and generalization. The epoch corresponding to the minimum validation loss was retained for final test evaluation, consistent with the early-stopping protocol described in Section 2.6.

To further assess result stability beyond the representative loss-curve example, the proposed pressure–vibration fusion model was trained and evaluated over ten repeated stratified random splits with different random seeds, following the same evaluation protocol used in the architecture-comparison and ablation experiments. The results summarized in Table 3 show that the model maintained stable performance across training, validation, and test sets over repeated runs. In particular, the relatively limited variation observed on the validation and test sets further supports the robustness of the proposed model under the present experimental setting, indicating that its performance did not depend strongly on a specific random partition of the dataset.

### 3.4. Confusion Matrix and Misclassification Analysis

**Figure 6:**
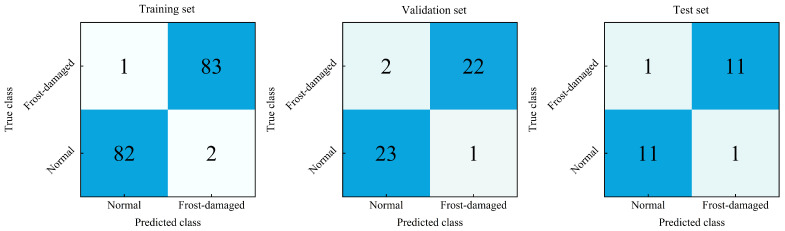
Confusion matrices of the proposed Transformer-based multimodal fusion model from a representative repeated-split run on the training set, validation set, and test set. Rows denote true classes and columns denote predicted classes.

To provide a more transparent view of class-wise prediction performance, the confusion matrices of the proposed Transformer-based multimodal fusion model from a representative repeated-split run on the training, validation, and test sets are shown in Figure 6. Rows denote true classes and columns denote predicted classes. In addition to the aggregate classification metrics reported above, these confusion matrices allow direct inspection of the distribution of correctly classified and misclassified normal and frost-damaged samples across different data splits.

Overall, the confusion matrices are consistent with the strong classification performance of the proposed model and show that most samples in both classes were correctly identified across the three datasets. As expected, the training-set confusion matrix exhibits the highest consistency with the learned decision boundary, whereas the validation and test matrices provide a more direct view of generalization performance on unseen samples. This pattern is also consistent with the repeated-run statistical results reported in Table 3.

On the validation and test sets, the small number of misclassified cases suggests that a limited subset of samples exhibited less separable tactile signatures under the present experimental setting. A brief examination of these errors indicates several possible causes. First, some fruits may have exhibited relatively mild or spatially heterogeneous frost injury, making the local tactile response measured during grasping less distinct from that of normal fruits. Second, because tactile perception depends on local contact conditions, variations in fruit pose, curvature, and contact state during grasping may have influenced the measured pressure and vibration responses. Third, biological variability among fruits may also have contributed to overlap in the multimodal tactile patterns of the two classes. These observations further indicate that borderline cases remain challenging and that future validation on larger and more diverse datasets would be beneficial.

Overall, the confusion matrices and misclassification analysis complement the aggregate evaluation metrics by providing a more detailed view of error distribution and possible sources of residual classification uncertainty.

### 3.5. Temporal Attribution Analysis

#### 3.5.1. Temporal Attribution Heatmap Visualization

**Figure 7:**
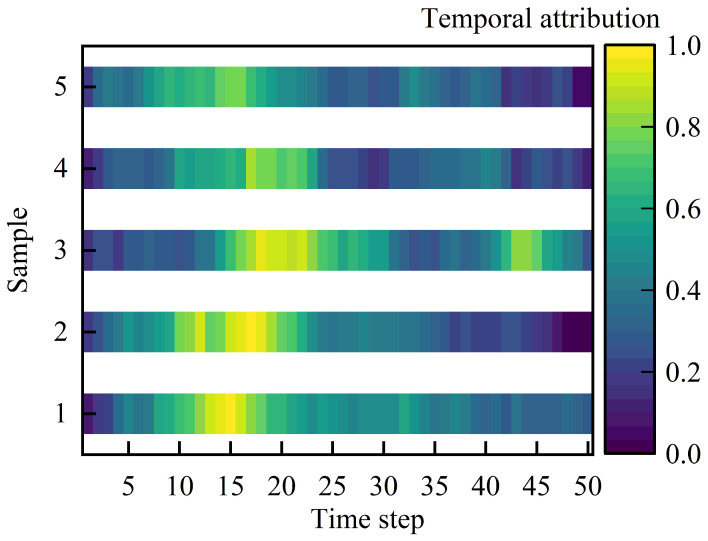
Temporal attribution heatmaps for five representative test samples across standardized time steps. Higher normalized values indicate stronger attribution within the fused temporal attribution profile described in Section 2.7.

Based on the fused normalized temporal attribution score ${\hat{s}}_{\mathrm{fused}}\left(t\right)$ defined in Section 2.7, the model’s relative emphasis across standardized time steps was visualized as a temporal attribution heatmap. For each sample, higher values of ${\hat{s}}_{\mathrm{fused}}\left(t\right)$ indicate stronger attribution, whereas lower values indicate weaker attribution. Figure 7 presents temporal attribution heatmaps for five representative test samples, providing an intuitive view of how attribution was distributed across the grasping sequence. The head-specific attention patterns within each modality branch showed broadly consistent temporal emphasis, with higher responses mainly appearing around the initial contact and early loading stages. Based on this consistency, the head-averaged attention representation was used to summarize the dominant temporal attribution pattern for each modality branch.

**Figure 8:**
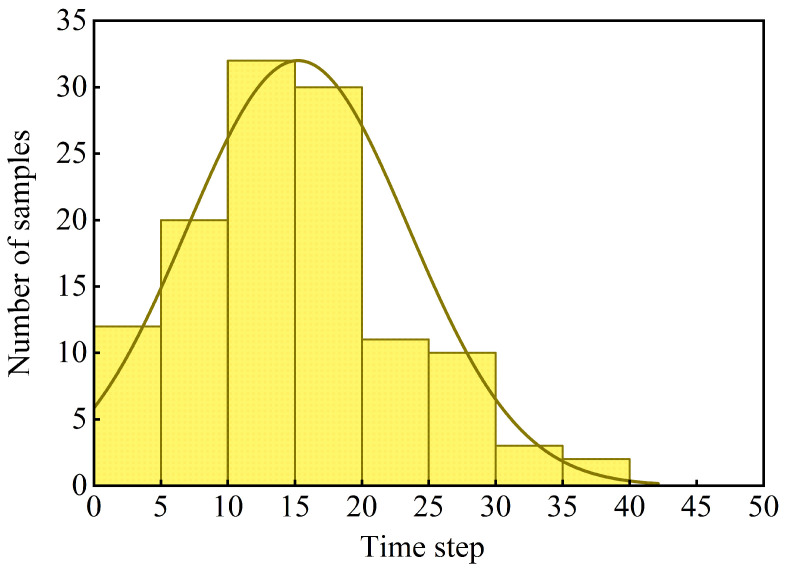
Distribution of peak-attribution time intervals across all samples, obtained from the fused normalized temporal attribution profiles.

To summarize the temporal locations that most frequently received the highest attribution, the peak time step $t^{*}=\arg\max_{t}{\hat{s}}_{\mathrm{fused}}\left(t\right)$ was computed for each sample. Following the procedure described in Section 2.7, peak time steps were grouped into time intervals and aggregated across all samples. The resulting distribution of peak-attribution intervals is shown in Figure 8.

#### 3.5.2. Interpretation of Temporal Attribution Patterns

The temporal attribution analysis provides additional insight into which phases of the grasping sequence are emphasized by the model [40]. As shown in Figure 8, peak-attribution time steps were most frequently concentrated in the early stage of grasping, suggesting that the model tended to place relatively greater emphasis on the initial contact phase. This stage corresponds to the onset of contact and load application, during which pressure distributions and vibration responses may exhibit pronounced transients that are potentially informative for distinguishing frost-damaged fruits from normal ones.

Following initial contact, attribution remained relatively elevated over a short subsequent interval, suggesting that the model also utilized tactile information during the early loading stage. This pattern is consistent with the possibility that the classifier relied not only on quasi-static contact outcomes, but also on transient tactile responses emerging as the fruit first deformed under load. Such an interpretation should be viewed as a post hoc attribution pattern rather than as direct evidence of a causal decision mechanism.

From a sensing perspective, this observation is also consistent with the multimodal design of the proposed framework. Pressure signals capture the redistribution of contact load during deformation, whereas vibration signals are sensitive to dynamic responses during contact onset and loading. The concentration of attribution in the early grasping stage therefore suggests that combining sustained and transient tactile cues may be valuable for early frost-damage detection. In addition, the fact that informative cues were concentrated in a relatively short interval implies that reliable screening may be achievable within a short grasp window, which is potentially favorable for future deployment in automated sorting systems.

Overall, the temporal attribution visualizations provide a time-resolved post hoc view of the model’s emphasis during grasping and complement the quantitative classification results by showing when discriminative tactile cues were more strongly highlighted.

## 4. Discussion

Early-stage frost damage is difficult to identify visually in robotic citrus sorting because internal tissue properties may change before obvious external symptoms appear. The effectiveness of the proposed method is consistent with the physical nature of frost injury: freeze–thaw damage disrupts tissue microstructure and mechanical integrity [41], causing frost-damaged fruits to respond differently from normal fruits under grasp-induced deformation. Accordingly, both pressure redistribution and vibration signals during grasping are influenced by these structural changes [42]. With contact-based sensing and a standardized grasping protocol, the model can therefore exploit mechanically meaningful differences that are not accessible from appearance alone.

The ablation results show that pressure provides stronger discriminative information than vibration when used alone, while the best performance is achieved by combining both modalities, indicating their complementarity. This is physically plausible because pressure captures distributed contact and loading patterns, whereas vibration reflects transient dynamic responses during deformation. The superior performance of the Transformer-based fusion model over the RNN, CNN, and LSTM baselines further indicates that frost-damage-related cues are strongly temporal. Consistently, the temporal attribution analysis suggested that the model tended to place relatively greater emphasis on the early stage of grasping, especially initial contact and early loading, which is consistent with the possibility that frost-damage-related differences are reflected in subtle transient mechanical responses. In this sense, the temporal attribution results provide a post hoc description of when the multimodal model places greater emphasis during grasping, and they further support the relevance of early contact dynamics for frost-damage discrimination.

Several limitations remain. The 6×6 pressure matrix has limited spatial resolution, which may restrict the capture of finer local mechanical variations during fruit deformation. In addition, the present study was based on a relatively limited dataset for deep-learning-based sequence modeling, and the current results should therefore be interpreted as evidence of method feasibility under controlled conditions rather than as definitive proof of broad deployment robustness. Although dropout regularization, early stopping, and repeated stratified random splits were used to reduce overfitting risk, the higher training performance compared with validation and test performance indicates that dataset-specific fitting cannot be fully excluded. Intra-class variability was partly constrained during sample preparation by selecting fruits with comparable maturity and external appearance and by applying standardized storage, frost-treatment, equilibration, and robotic grasping procedures. This controlled design helped reduce variations unrelated to frost injury, but it also narrowed the variability represented in the dataset.

The present study also considered only binary classification and was restricted to a single cultivar with laboratory-induced frost injury. In each repeated split, the test samples were held out from model training and validation, providing internal evidence of generalization to unseen samples from the same experimental campaign. Although this design enabled controlled evaluation of the proposed sensing-and-learning framework, it only partially represents the biological and operational variability encountered in practical postharvest applications. Such variability may arise from differences among citrus genotypes and cultivars, fruit-to-fruit heterogeneity within a genotype, orchard and harvest conditions, naturally occurring frost damage, and changes in contact state, pose, and throughput during real sorting operations. Future validation should therefore extend to a larger number of citrus genotypes, broader multi-batch sample collections, naturally frost-damaged fruits, more diverse fruit-size and maturity distributions, and online or semi-online sorting scenarios, in order to better assess model robustness, borderline cases, and generalizability under practical postharvest conditions.

## 5. Conclusions

This study proposed a robotic pressure–vibration multimodal tactile sensing approach for early detection of frost-damaged citrus fruits. Experimental results across repeated stratified random-split evaluations demonstrated that the proposed method achieved strong classification performance. In addition, pressure–vibration fusion outperformed either single modality alone, and attention-based temporal attribution analysis suggested that informative tactile cues were more strongly emphasized during the initial contact and early loading stages of grasping.

These findings support the feasibility of grasp-based robotic frost-damage detection for citrus sorting under the present controlled experimental setting and suggest that early transient tactile responses provide useful information for discrimination between normal and frost-damaged fruits. Future work will focus on improving pressure sensing resolution, extending the framework to multi-level frost-damage severity assessment, and validating the method across additional cultivars, naturally damaged samples, and online sorting conditions. Further studies should also examine broader sample sets and repeated experiments to better assess model robustness and generalizability under practical postharvest conditions.

## Figures and Tables

**Table 1:** Performance comparison of different network architectures over 10 repeated stratified random splits (training:validation:test = 7:2:1). Results are reported as mean ± standard deviation.

Network Architecture	Accuracy	Class	Precision	Recall	F1-Score
RNN	0.842±0.029	Normal	0.831±0.038	0.864±0.046	0.846±0.030
		Frost-damaged	0.857±0.043	0.817±0.051	0.835±0.033
CNN	0.818±0.032	Normal	0.809±0.041	0.838±0.048	0.822±0.034
		Frost-damaged	0.831±0.046	0.799±0.056	0.813±0.037
LSTM	0.861±0.027	Normal	0.845±0.035	0.892±0.040	0.867±0.027
		Frost-damaged	0.883±0.038	0.832±0.047	0.856±0.031
Transformer	0.931±0.019	Normal	0.934±0.024	0.919±0.029	0.926±0.020
		Frost-damaged	0.928±0.028	0.943±0.026	0.935±0.021

**Table 2:** Classification performance under different input-modality configurations over 10 repeated stratified random splits (training:validation:test = 7:2:1). Results are reported as mean ± standard deviation. Precision, recall, and F1-score are reported for each class.

Input Modality	Accuracy	Class	Precision	Recall	F1-Score
Vibration (single modality)	0.829±0.031	Normal	0.847±0.039	0.803±0.052	0.824±0.034
		Frost-damaged	0.816±0.044	0.857±0.048	0.835±0.032
Pressure (single modality)	0.892±0.026	Normal	0.918±0.031	0.864±0.037	0.890±0.026
		Frost-damaged	0.871±0.036	0.921±0.034	0.895±0.027
Pressure + Vibration (fusion)	0.931±0.019	Normal	0.934±0.024	0.919±0.029	0.926±0.020
		Frost-damaged	0.928±0.028	0.943±0.026	0.935±0.021

**Table 3:** Statistical robustness of the proposed pressure–vibration fusion model over ten repeated stratified random splits with different random seeds.

Dataset	Accuracy	Class	Precision	Recall	F1-Score
Training	0.982±0.005	Normal	0.983±0.006	0.980±0.007	0.981±0.005
		Frost-damaged	0.980±0.007	0.984±0.006	0.982±0.005
Validation	0.948±0.014	Normal	0.951±0.017	0.944±0.019	0.947±0.015
		Frost-damaged	0.946±0.018	0.951±0.016	0.948±0.014
Test	0.931±0.019	Normal	0.934±0.024	0.919±0.029	0.926±0.020
		Frost-damaged	0.928±0.028	0.943±0.026	0.935±0.021

## Data Availability

The data presented in this study are available on request from the corresponding author.

## References

[1] FAOSTAT (2024). Food and Agriculture Organization of the United Nations (FAO). https://www.fao.org/faostat/en/#home.

[2] Rahmanian A., Mireei S.A., Sadri S., Gholami M., Nazeri M. (2020). Application of Biospeckle Laser Imaging for Early Detection of Chilling and Freezing Disorders in Orange. Postharvest Biol. Technol..

[3] Slaughter D., Obenland D., Thompson J., Arpaia M., Margosan D. (2008). Non-Destructive Freeze Damage Detection in Oranges Using Machine Vision and Ultraviolet Fluorescence. Postharvest Biol. Technol..

[4] Li D., Zhu Z., Sun D.W. (2018). Effects of Freezing on Cell Structure of Fresh Cellular Food Materials: A Review. Trends Food Sci. Technol..

[5] Liu N., Li X., Zhao P., Zhang X., Qiao O., Huang L., Guo L., Gao W. (2021). A Review of Chemical Constituents and Health-Promoting Effects of Citrus Peels. Food Chem..

[6] Hu W., Xiong J., Liang J., Xie Z., Liu Z., Huang Q., Yang Z. (2023). A Method of Citrus Epidermis Defects Detection Based on an Improved YOLOv5. Biosyst. Eng..

[7] Wang D., Zhang M., Jiang Q., Mujumdar A.S. (2024). Intelligent System/Equipment for Quality Deterioration Detection of Fresh Food: Recent Advances and Application. Foods.

[8] Tian S., Wang S., Xu H. (2022). Early Detection of Freezing Damage in Oranges by Online Vis/NIR Transmission Coupled with Diameter Correction Method and Deep 1D-CNN. Comput. Electron. Agric..

[9] Wang Q., Tu Y., Xu W., Zhang J., Knoll A., Zhou M., Ying Y. (2025). Towards Damage-Less Robotic Fragile Fruit Grasping: A Systematic Review on System Design, End Effector, and Visual and Tactile Feedback. J. Field Robot..

[10] Luo S., Lepora N.F., Yuan W., Althoefer K., Cheng G., Dahiya R. (2025). Tactile Robotics: An Outlook. IEEE Trans. Robot..

[11] Chauhan A., Brouwer B., Westra E. (2022). Robotics for a Quality-Driven Post-Harvest Supply Chain. Curr. Robot. Rep..

[12] Zou S., Picella S., De Vries J., Kortman V.G., Sakes A., Overvelde J.T.B. (2024). A Retrofit Sensing Strategy for Soft Fluidic Robots. Nat. Commun..

[13] Jin L., Wang Z., Tian S., Feng J., An C., Xu H. (2023). Grasping Perception and Prediction Model of Kiwifruit Firmness Based on Flexible Sensing Claw. Comput. Electron. Agric..

[14] Chen Y., Lin J., Du X., Fang B., Sun F., Li S. Non-Destructive Fruit Firmness Evaluation Using Vision-Based Tactile Information. Proceedings of the 2022 International Conference on Robotics and Automation (ICRA).

[15] Park S., Hwang D. (2021). Softness-Adaptive Pinch-Grasp Strategy Using Fingertip Tactile Information of Robot Hand. IEEE Robot. Autom. Lett..

[16] Blanes C., Mellado M., Beltrán P. (2016). Tactile Sensing with Accelerometers in Prehensile Grippers for Robots. Mechatronics.

[17] Dobashi Y., Yao D., Petel Y., Nguyen T.N., Sarwar M.S., Thabet Y., Ng C.L.W., Scabeni Glitz E., Nguyen G.T.M., Plesse C. (2022). Piezoionic Mechanoreceptors: Force-Induced Current Generation in Hydrogels. Science.

[18] Park K., Yuk H., Yang M., Cho J., Lee H., Kim J. (2022). A Biomimetic Elastomeric Robot Skin Using Electrical Impedance and Acoustic Tomography for Tactile Sensing. Sci. Robot..

[19] Chun S., Kim J.S., Yoo Y., Choi Y., Jung S.J., Jang D., Lee G., Song K.I., Nam K.S., Youn I. (2021). An Artificial Neural Tactile Sensing System. Nat. Electron..

[20] Wang Z., Tang Y., Yao P., Chen Y., Luo J., Xue W., Ma Y., Wang J. (2026). Bioinspired Flexible Tactile Sensors for Smart Soft Robotics. ACS Appl. Mater. Interfaces.

[21] Lu Y., Kong D., Yang G., Wang R., Pang G., Luo H., Yang H., Xu K. (2023). Machine Learning-enabled Tactile Sensor Design for Dynamic Touch Decoding. Adv. Sci..

[22] Gu H., Lu B., Gao Z., Wu S., Zhang L., Xie L., Yi J., Liu Y., Nie B., Wen Z. (2024). A Battery-free Wireless Tactile Sensor for Multimodal Force Perception. Adv. Funct. Mater..

[23] Xu P., Zhu X., Clifton D.A. (2023). Multimodal Learning with Transformers: A Survey. IEEE Trans. Pattern Anal. Mach. Intell..

[24] Vaswani A., Shazeer N., Parmar N., Uszkoreit J., Jones L., Gomez A.N., Kaiser L., Polosukhin I., Guyon I., Luxburg U.V., Bengio S., Wallach H., Fergus R., Vishwanathan S., Garnett R. (2017). Attention Is All You Need. Proceedings of the Advances in Neural Information Processing Systems 30 (Nips 2017).

[25] Sun L., Lian Z., Liu B., Tao J. (2024). Efficient Multimodal Transformer with Dual-Level Feature Restoration for Robust Multimodal Sentiment Analysis. IEEE Trans. Affect. Comput..

[26] Tan E.S., Slaughter D.C., Thompson J.F. (2005). Freeze Damage Detection in Oranges Using Gas Sensors. Postharvest Biol. Technol..

[27] Zhang Y., Wang Z. (2025). Review of Robotic Grippers for High-Speed Handling of Fragile Foods. Adv. Robot..

[28] Mulholland B.J., Panesar P.S., Johnson P.H. (2024). The Adoption of Robotics in Pack Houses for Fresh Produce Handling. J. Hortic. Sci. Biotechnol..

[29] Tee B.C.K., Chortos A., Berndt A., Nguyen A.K., Tom A., McGuire A., Lin Z.C., Tien K., Bae W.G., Wang H. (2015). A Skin-Inspired Organic Digital Mechanoreceptor. Science.

[30] Sharma Y., Ferreira P., Justham L. (2024). Hardness Classification Using Cost-Effective off-the-Shelf Tactile Sensors Inspired by Mechanoreceptors. Electronics.

[31] Li F., Wang R., Song C., Zhao M., Ren H., Wang S., Liang K., Li D., Ma X., Zhu B. (2021). A Skin-Inspired Artificial Mechanoreceptor for Tactile Enhancement and Integration. ACS Nano.

[32] Jo Y., Lee Y., Kwon J., Kim S., Ryu G., Yun S., Baek S., Ko H., Jung S. (2025). 3D Active-Matrix Multimodal Sensor Arrays for Independent Detection of Pressure and Temperature. Sci. Adv..

[33] Niu J., Zhang C., Chen X., Ma C., Chen L., Tong C. (2019). A Novel Helmet Fitness Evaluation Device Based on the Flexible Pressure Sensor Matrix. Sensors.

[34] Yeom H.I., Kim J., Jeon G.J., Kim J., Park S.H.K. (2023). Active-Matrix Driven Flexible Pressure Sensor Array Using Oxide Thin-Film Diode. IEEE Electron Device Lett..

[35] Fu C., Gao C., Zhang W. (2024). RUL Prediction for Piezoelectric Vibration Sensors Based on Digital-Twin and LSTM Network. Mathematics.

[36] Wan J., Liu J., Zhou J., Lai Z., Shen L., Sun H., Xiong P., Min W. (2023). Precise Facial Landmark Detection by Reference Heatmap Transformer. IEEE Trans. Image Process..

[37] Abnar S., Zuidema W. Quantifying Attention Flow in Transformers. Proceedings of the 58th Annual Meeting of the Association for Computational Linguistics.

[38] Chefer H., Gur S., Wolf L. Transformer Interpretability beyond Attention Visualization. Proceedings of the 2021 IEEE/CVF Conference on Computer Vision and Pattern Recognition (CVPR).

[39] Liao Z., Yang Z., Huang P., Pang N., Zhao X. (2023). Multi-Model Fusion-Based Hierarchical Extraction for Chinese Epidemic Event. Data Sci. Eng..

[40] Phan H., Mikkelsen K., Chen O.Y., Koch P., Mertins A., De Vos M. (2022). SleepTransformer: Automatic Sleep Staging with Interpretability and Uncertainty Quantification. IEEE Trans. BioMed. Eng..

[41] Dalvi-Isfahan M., Jha P.K., Tavakoli J., Daraei-Garmakhany A., Xanthakis E., Le-Bail A. (2019). Review on Identification, Underlying Mechanisms and Evaluation of Freezing Damage. J. Food Eng..

[42] Wibowo S., Buvé C., Hendrickx M., Van Loey A., Grauwet T. (2018). Integrated Science-Based Approach to Study Quality Changes of Shelf-Stable Food Products during Storage: A Proof of Concept on Orange and Mango Juices. Trends Food Sci. Technol..

